# Normative 3D opto-electronic stereo-photogrammetric posture and spine morphology data in young healthy adult population

**DOI:** 10.1371/journal.pone.0179619

**Published:** 2017-06-22

**Authors:** Moreno D'Amico, Edyta Kinel, Piero Roncoletta

**Affiliations:** 1 SMART Lab (Skeleton Movement Analysis & Advanced Rehabilitation Technologies) Bioengineering & Biomedicine Company Srl, Pescara, Italy; 2 Department of Rheumatology and Rehabilitation, Clinic of Rehabilitation, University of Medical Sciences, Poznan, Poland; Queensland University of Technology, AUSTRALIA

## Abstract

Design: Observational cross-sectional study. The current study aims to yield normative data: i.e., the physiological standard for 30 selected quantitative 3D parameters that accurately capture and describe a full-skeleton, upright-standing attitude. Specific and exclusive consideration was given to three distinct categories: postural, spine morphology and pelvic parameters. To capture such 3D parameters, the authors selected a non-ionising 3D opto-electronic stereo-photogrammetric approach. This required the identification and measurement of 27 body landmarks, each specifically tagged with a skin marker. As subjects for the measurement of these parameters, a cohort of 124 asymptomatic young adult volunteers was recruited. All parameters were identified and measured within this group. Postural and spine morphology data have been compared between genders. In this regard, only five statistically significant differences were found: pelvis width, pelvis torsion, the “lumbar” lordosis angle value, the lumbar curve length, and the T12-L5 anatomically-bound lumbar angle value. The “thoracic” kyphosis mean angle value was the same in both sexes and, even if, derived from skin markers placed on spinous processes it resulted in perfect agreement with the X-ray based literature. As regards lordosis, a direct comparison was more difficult because methods proposed in the literature differ as to the number and position of vertebrae under consideration, and their related angle values. However, when the L1 superior–L5 inferior end plate Cobb angle was considered, these results aligned strongly with the existing literature. Asymmetry was a standard postural-spinal feature for both sexes. Each subject presented some degree of leg length discrepancy (LLD) with μ = 9.37mm. This was associated with four factors: unbalanced posture and/or underfoot loads, spinal curvature in the frontal plane, and pelvis torsion. This led to the additional study of the effect of LLD equalisation influence on upright posture, relying on a sub-sample of 100 subjects (51 males, 49 females). As a result of the equalisation, about 82% of this sub-sample showed improvement in standing posture, mainly in the frontal plane; while in the sagittal plane less than 1/3 of the sub-sample showed evidence of change in spinal angles. A significant variation was found in relation to pelvis torsion: 46% of subjects showed improvement, 49% worsening. The method described in study presents several advantages: non-invasive aspect; relatively short time for a complete postural evaluation with many clinically useful 3D and 2D anatomical/biomechanical/clinical parameters; analysis of real neutral unconstrained upright standing posture.

## Introduction

Spine and posture disorders are topics of great interest in biomechanical research and in a variety of clinical fields. In recent decades as a result of both financial and clinical issues spinal and postural functional assessment has progressively gained importance for clinical practice [1]. The financial issues relate primarily to the growing pressure exerted by insurance companies and health institutions for an objective assessment of impairments related to ill-health/injury, a source of large and constantly increasing health and social care costs. The major categories of ill-health/injury under consideration are those involving the spine [2] and back pain, degenerative neural disorders/stroke [3] and falls in the elderly [4]. The latter in particular arises from the involvement of postural and trunk malfunction in the pathogenesis of a range of musculoskeletal disturbances [2,5].

From all the above it appears evident that quantitative posture and spine morphology analysis is crucial for clinical assessments in physical medicine and rehabilitation [1]. It is important to establish the relationship between postural balance and anthropometric measurements for determining reference values in normal and pathological conditions [6]. This framework has to be taken into account in designing and developing treatment programs in rehabilitation, planning of orthopaedic surgical procedures [5] and monitoring the progression of pathology and/or treatment outcomes [7,8].

In general, human posture can be described as the attitude of the body when sitting, walking, or standing. Posture is a dynamic event, even in relation to the simple neutral standing-erect attitude. In reality, human posture is the outcome of a very complex neuromotor-biomechanical system and can be defined as “a continuously evolving dynamic event” expressed as a position of the body maintained in space for some time under the continuous control of the central nervous system (CNS) [9,10]. Being a dynamic event, this position is characterised by an “*equilibrium status*” and the intrinsic variability in term of oscillations around this status. The intrinsic variability is strictly connected to any given mental and physiological status (healthy, pathological, voluntarily maintained, fatigued, under physical and/or psychological stress, etc.) [10,11]. Moreover, it can be affected by different factors including familial physical factors, anatomical structural impairments, postural habits and work activities.

Changes in postural attitude, and the variability of such changes, are therefore very important parameters as a means of quantifying the “good” functional movements of a human body system as well as of CNS [10,11]. From an instrumental point of view, obtaining such quantitative functional evaluation is a challenging matter especially when full-skeleton posture, including the 3D-shape of the spine, needs to be described. The development of diagnostic technologies based on image-processing (e.g., digital X-ray, digital 3D stereo X-ray reconstruction, CAT scans and MRI) has provided a significant improvement in obtaining increasingly accurate and detailed information about anatomical structures, especially in the evaluation of spine-related pathologies. However, except for dynamic X-ray and the very recent dynamic MRI, no single one of these techniques is able to provide information about the functional state of the vertebral column and related patient posture [12,13].

On the other hand, while two-dimensional (2D) images are still widely used in clinical examination their technical limitations create the risk of evaluation errors and represent a major source of variability that may conceal the actual geometrical relationship between anatomical structures [14]. Such limitations include their potentially harmful ionizing effects, reliance on the “single shot” method, the presence of image noise, distinctive characteristics of imaging techniques and the variable positioning of the patient during image acquisition.

In recent decades, several instrumental non-invasive techniques have been developed to overcome the limitations of manual and “single shot” radiological methods. Unfortunately, many of these techniques, such as electro-goniometric and/or flexicurve devices, still embody certain limitations, such as the inability to perform simultaneous 3D measurements through the whole spine and/or the requirement that the subject maintains a specific postural attitude [1,15,16]. Some interesting low-cost photographic methods have also emerged, with promising results but corresponding limitations: the single shot approach, weak calibration procedures, and lack of genuinely instantaneous 3D posture measurement (the coronal and sagittal plane are not recorded at the same moment in time) [17–22].

Furthermore, even the promising rastereography back-surface measurement technique raises questions and doubts, particularly regarding discrepancies with respect to X-ray techniques in the coronal plane, that require further clarification [15,23]. Given the restrictions above, it is crucial that innovative methods and non-ionising technologies be developed in order to capture and quantify 3D posture and spine morphology, for the purpose of clinical assessment in physical medicine and rehabilitation [1].

In this context, opto-electronic stereo-photogrammetric measurement offers a meaningful solution for the capture of functional information necessary for addressing clinical problems in rehabilitation medicine: specifically, it quantitatively captures both static and dynamic body postural expressions using 3-dimensional (3D) imaging of both posture and spinal shape. It can achieve this for a static-erect posture as well as related oscillations and additionally eventual behaviour of body segments during movement. Moreover, given that this method has no harmful effects, it provides a “natural” approach for both capturing and monitoring the progression of pathology and/or treatment outcomes.

The use of stereo-photogrammetry-based postural and movement analysis, and its contribution to scientific knowledge is increasingly being reported in the literature exploring different original approaches [1,12,13,24–40]. Extending this prior research, we will focus in this paper on a special protocol and elaboration technique that allows us to construct a complete 3D parametric biomechanical model of the human skeleton. The model contains detailed information for the 3D reconstruction of spinal shape, providing a thorough description of standing posture and fulfilling all the necessary requirements (above) for human posture measurement [12,13,33–39,41]. This approach has been widely tested and used on more than one thousand of patients with different orthopaedic and neurological disorders, both in clinical rehabilitation centres and hospitals [10–13,33–39,41–48]

The clinical use of these new technological tools, of course, necessitates the establishment of new standards and parameters for reference values in normal and pathological conditions; to date, the primary reference point has been normative data derived from X-ray or classical clinical analysis. There now exists the logical necessity for new 3D stereo-photogrammetric reference data, and for the comparison of such data with existing reference standards. A limited set of preliminary normative data has previously been published [47] in relation to the approach under consideration. In this paper, the authors aim to present a fuller and more comprehensive set of normative data for the definition of basal 3D quantitative postural, spine morphology and pelvic parameters, reflecting an erect-standing posture in a young healthy adult population, by using a non-ionising 3D opto-electronic stereo-photogrammetric approach.

## Materials and methods

### Design

The research presented here is an observational cross-sectional study. A cohort of 124 asymptomatic young adult university students and workers, all volunteers, was measured in order to establish the baseline normative data described above.

### Study population

The cohort population had the following characteristics: Caucasian males and females in the age range 18–35 years; subjects not undertaking sports activity at agonistic level; self-declaration of no orthopaedic or neurologic problems; no history of musculoskeletal system injury or surgery; Body Mass Index (BMI) within normal range: 18.50 = /< BMI = /< 24.99 as established by the World Health Organisation (WHO) [49].

The study was carried out in conformity with the Declaration of Helsinki and, given the innocuous quality of the measurement process, the focus on merely postural characteristics of healthy subjects, strict data privacy protection (data are published anonymously and only in statistical form) and the voluntary participation of all the subjects, the research was formally approved by the Local Medical Ethics Committee and confirmed by BioEthics Committee Poznan University of Medical Sciences (n. 1255/16). After being fully informed about the risk-free nature of the evaluation and the aims of the research, each subject voluntarily signed a consent form.

Data collection took place between March and December 2015. Population characteristics are summarized in Table 1.

**Table 1:** Sample population characteristics: Total 124 healthy young adults.

Population Characteristics	Female (n = 57)		Male (n = 67)		t-Test Female vs Male
	Range	Mean± SD*	Range	Mean± SD*	
**Age [years]**	19–34	23.5±3.2	20–35	24.9±3.9	NS**
**Height [cm]**	155–175	163.9±5.3	164–190	178.3±6.7	P<0.001
**Weight [kg]**	40–71	56.1±7.0	50–90	71.8±8.6	P<0.001
**BMI [kg/m**^**2**^**]**	15.6–24.8	20.8±2.0	18.6–24.9	22.5±1.6	NS**

* SD *= Standard Deviation*

**NS *= Not Significant*

### Instrumentation and measurement processing

**Fig 1:**
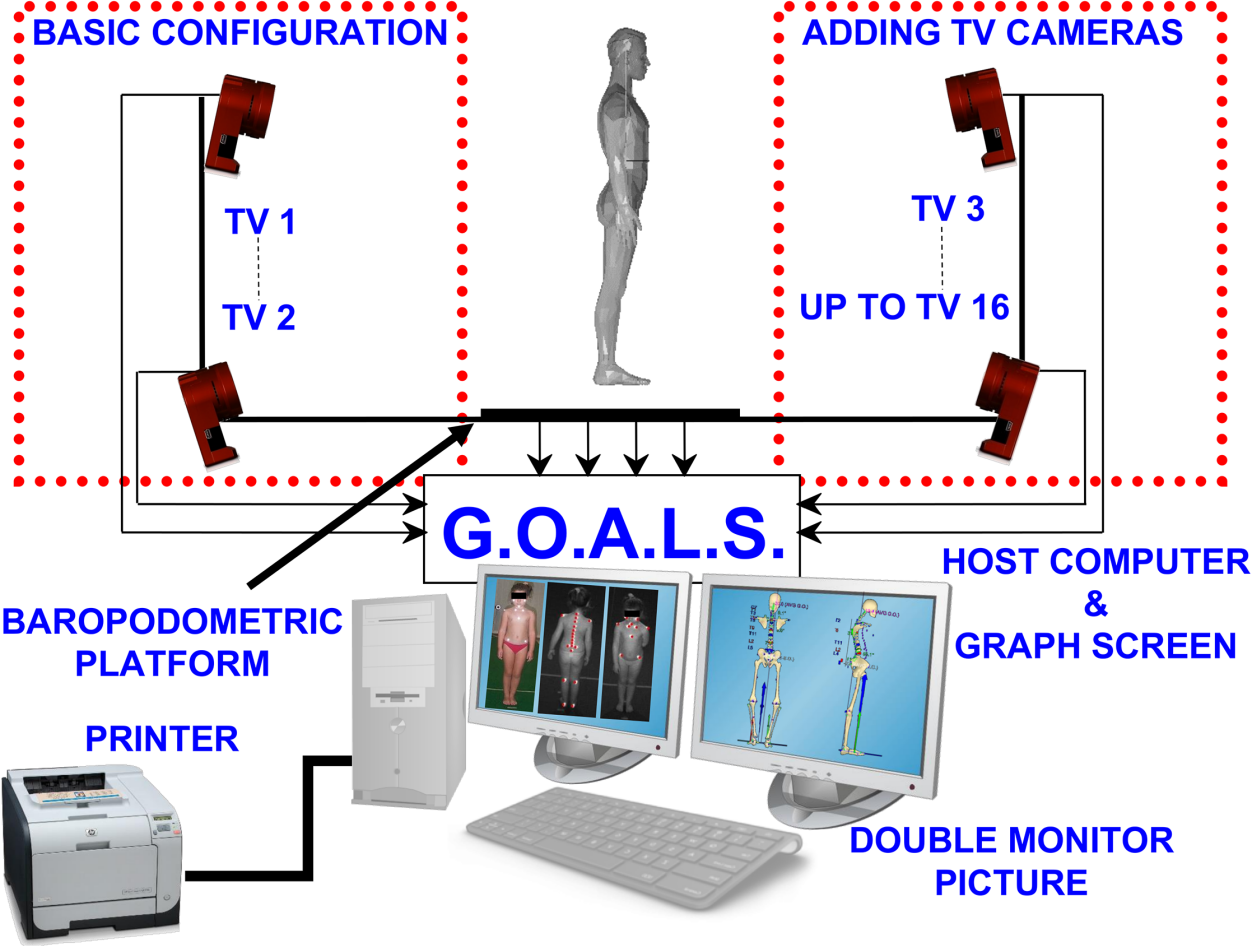
The experimental set-up used for 3D posture analysis: GOALS system and baropodometric platform configuration.

Our experimental recordings are based on a brand new 6TVC G.O.A.L.S. (Global Opto-electronic Approach for Locomotion & Spine) (Bioengineering & Biomedicine Company S.r.l. Italy) stereo-photogrammetric opto-electronic system derived from Optitrack System (NaturalPoint Inc. USA). In addition, we used a baropodometric platform (Zebris Gmbh, Germany) to synchronously measure foot-pressure maps as well as the vertical forces exerted on each of the subject’s feet while standing (Fig 1).

This is a general methodology which can be applied indifferently to any stereo-photogrammetric recording system, provided that the latter is able to supply all the required landmarks three-dimensional coordinates. The underlying assumption is that the human body can be modelled as a collection of rigid segments connected by ball or hinge joints. For data processing, the essential information being sought is the measurement of posture (and eventually of movements) of the full 3D skeleton, spinal shape included. Subject-specific models have to be built upon measured data to assess kinematic and dynamic variables of posture and movement.

With this goal in mind, an initial project [12] was undertaken in the mid-1990s to create a complete and accurate 3D biomechanical model of the human skeleton, with particular attention to the precise reproduction of spinal detail. This was achieved by merging different segmental biomechanical models presented in the literature [12]. The model was conceived in parametric form in order to enable scaling of the individual characteristics of any subject. This would be achieved by fitting the 3D anthropometric size of a given skeletal segment, to 3D opto-electronic measurements of the corresponding body landmarks labelled by passive markers [12].

Particular care and attention have been devoted to devising and refining the parameters of this proposed 3D model of the human skeleton. The accuracy and precision of the model rely on anatomical findings (cadaver dissections, in vivo X-ray and gamma ray measurements, and parametric regression equations) [50–55] as well as a variety of signal-processing procedures and optimization methods described in the literature [12,13,33–37,41,42,56–58]. This complete 3D parametric biomechanical model of the human skeleton is now available as a commercial software package named ASAP 3D Skeleton Model (Bioengineering & Biomedicine Company S.r.l. Italy).

This sophisticated software package has been used for all data processing required for the extraction of quantitative postural parameters as presented in this paper. The model, as developed, can work at different levels of complexity depending on the purposes and requirements of the analysis. It is possible to increase the degree of detail for parametric scaling by obtaining additional anthropometric measurements relative to specific anatomical segments (e.g., lower limbs) if there is an interest in one or more specific characteristics of the body. To this end, various protocols involving different body labelling have been established for different analytical purposes [12,13,34–37].

**Fig 2:**
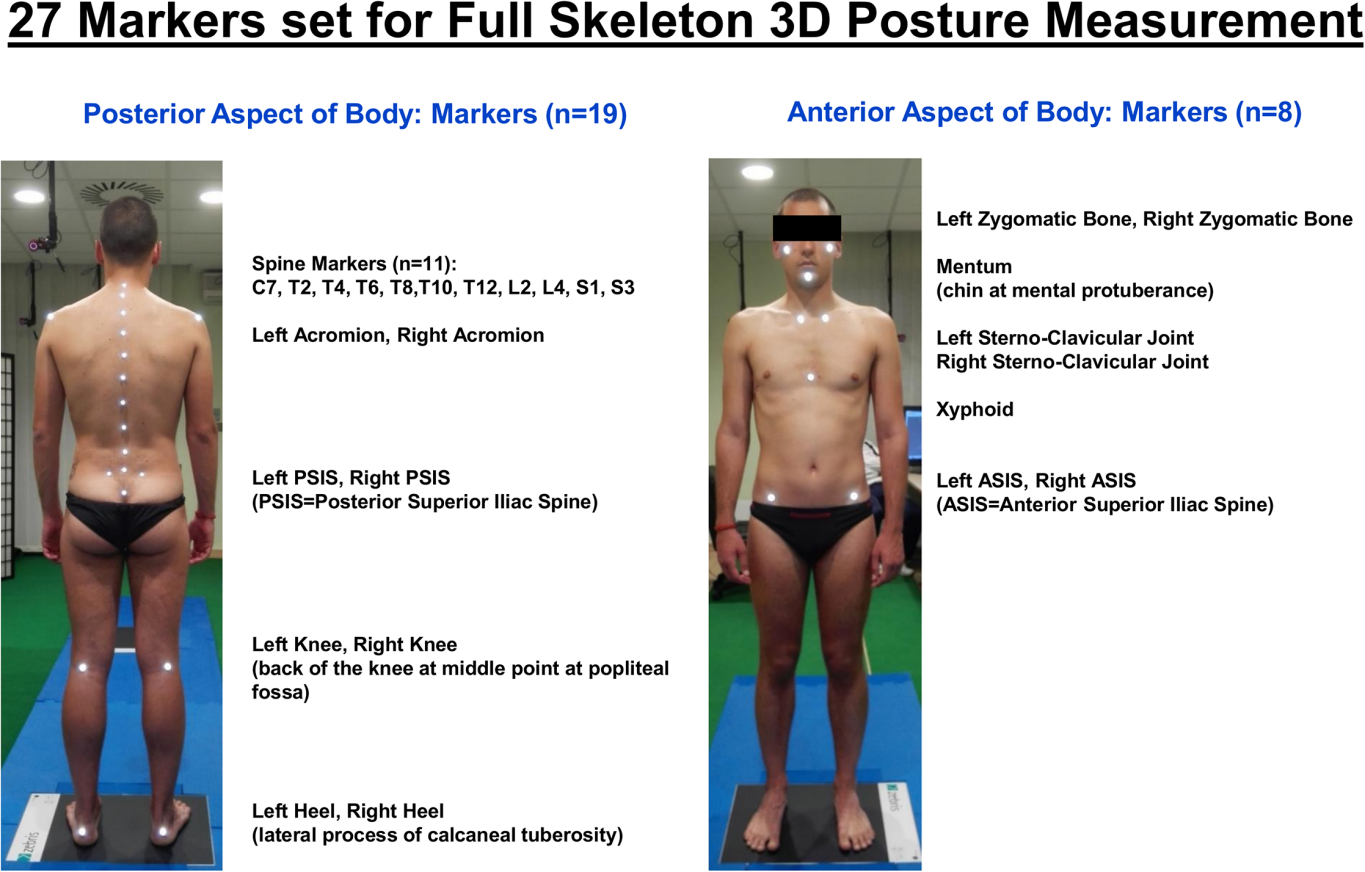
Protocol for 3D posture analysis: List of 27 anatomical landmarks identified by palpation.

**Fig 3:**
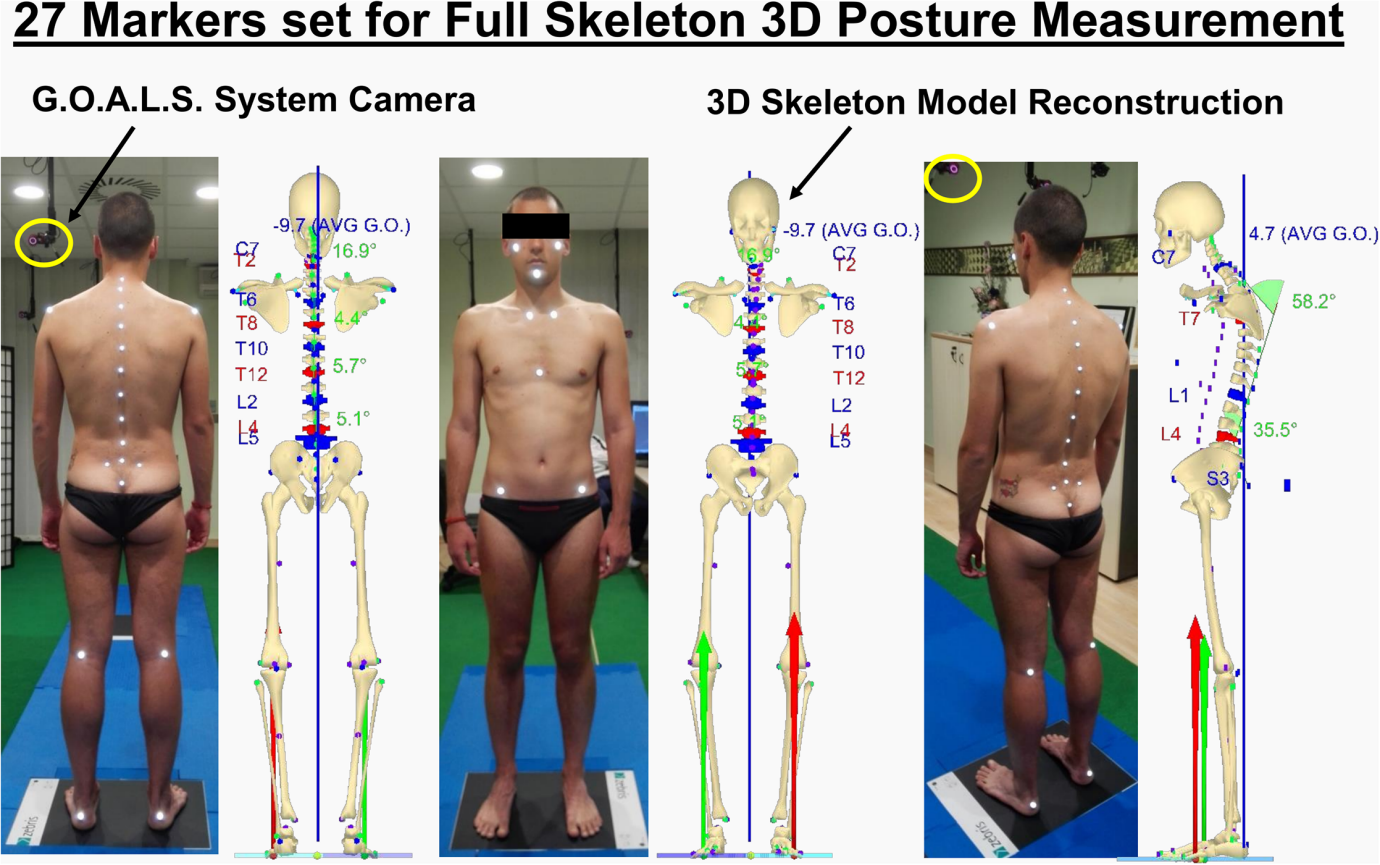
An example of full 3D skeleton reconstruction.

The original protocol, which has been developed for analysis of human posture and spine-related pathologies (scoliosis, back pain, etc.) has been devised and tested extensively in the clinical environment [10,35,37,48]. It is based on 27 skin markers accurately positioned and glued on the human body: a comprehensive list of these anatomical landmarks (as identified by palpation) is given in Fig 2. Using the 3D measurements of these 27 markers, a 3D quantitative delineation of individual body posture can be computed and reproduced, taking into account the postural attitude of the head, trunk, pelvis and legs (the upper limbs and ribs are not considered) as well as the shape of the spine in 3D at each metameric level. An example of a full 3D skeleton reconstruction, obtained via opto-electronic stereo-photogrammetric measurement, can be found in Fig 3.

Once the reconstruction of the skeleton has been accurately achieved, useful quantitative biomechanical parameters can be extracted both directly and indirectly from the measurements. Taking the pelvis as an example, factors such as orientation, scaling and eventual torsion can be assessed directly from measurements of ASIS and PSIS (Anterior and Posterior Iliac Spine) positions. Additionally, by using regression equations (as presented in the literature [50]) the same anatomical landmarks can provide the basis for indirect measurements of Hip Joint Centre (HJC) positions and of pelvis width (PW) defined as their Inter distance (Inter Hip Joint Centers Distance: IHJCD).

### Acquisition protocol

The calibrated acquisition volume was 3m x 3m x 2m. The calibration provided by the GOALS-Optitrack system is of the wand type, based on geometrical epipolar constraint [59–62] that allows the estimation of focal lengths, principal points and lens distortions of the cameras without the use of any additional device. Therefore, the computerised calibration of the internal and external parameters is enabled simply by surveying and capturing the 3D movement of a rigid bar (wand) with three markers at a known distance, in motion inside the working volume. This approach overcomes working volume limits of previously proposed methods such as Direct Linear Transformation (DLT) [63] and provides far better results [64]; see also Chiari et al. [65] for review. The final mean 3D stereo-photogrammetric error was limited to a range of 0.3–0.4mm throughout the entire working volume.

All subjects in this research were given a face-to-face interview and a thorough clinical postural examination before their session of postural measurement. To ensure consistency, all the interviews and physical examinations were conducted by a single qualified physiotherapist with 14 years experience (the second named author) in order to confirm the subjects were free from any orthopaedic/neurological disorder. In case of any doubt, the intervention of one or more of the physicians belonging to the Local Medical Committee was asked.

Furthermore, in order to reduce potential postural effects resulting from circadian rhythms, all measurements were taken between 12 noon and 7.00 pm. The subjects were asked to avoid any intensive training and/or hard physical activity before the postural assessment.

The assessment/measurement session aimed to fully capture and record the subject’s neutral standing posture, with the upper arms relaxed along the side of the body and eyes looking directly ahead in the horizontal plane. Such posture has been defined as Indifferent Orthostasis (IO). For all subjects, marker positioning was performed by a single operator with more than 20 years experience. Each marker-positioning session was of approximately 10 minutes duration, after which the subject was asked to sit for a few minutes.

Soon after the subject was asked to keep an Indifferent Orthostasis standing with both feet on a baropodometric platform.

Each static postural attitude was considered to have been accurately recorded when at least five acquisitions, each of two seconds duration, were captured. Given the data acquisition rate of 120Hz (for the opto-electronic device used in this research), this means that for each static postural stance a minimum of 1200 measurements was averaged [10,12,35,48,49].

After 24 subjects had been measured for IO posture, a provisional early analysis showed an unexpected feature in the quantitative data: every subject was presenting with some degree of leg length discrepancy (LLD); in other words a difference in right vs. left hip centre heights as assessed by Anterior and Posterior Iliac Spine (ASIS and PSIS) landmark positions [50]). LLD was associated with some degree of pelvic tilt in the coronal plane and very often with pelvis torsion in the sagittal plane; i.e., the inter-ASIS height differences and the inter-PSIS height differences were often very dissimilar. A pelvic torsion is usually defined as an intrasegmental pelvic pattern in which one ilium is more tilted towards the anterior than the other [66]. In the literature LLD is reported as relatively common, affecting up to 90% of the population with an average value of 5.2 ± 4.1mm as measured by highly precise radiographic millimetric methods [66].

As a result of this early finding, it was decided to expand the investigation to include the effect (if any) that could be achieved on neutral standing posture by equalisation of the lower limbs. Therefore, a new element was added to the protocol as follows: a suitable wedge would be placed under the subject’s foot as a corrective, to equalise any found LLD; this was called “Wedge-Corrected Orthostasis” (WCO). An additional measurement was then conducted (with the wedge in place) for each of the remaining 100 subjects in the original cohort, and the data collected for analysis. In order to take into account both “anatomical” and “functional” LLD [66] it was decided that the optimal size for the corrective wedge was the degree of thickness that provided the best outcome for the individual subject in relation to frontal-plane postural parameters (see below) with a particular focus on pelvic parameters. In a small number of subjects, the equalisation did not produce an overall improvement of the frontal-plane postural parameters. In these cases, the authors judged that the optimal corrective-wedge thickness should be that which provided the best equalisation as regards the posterior superior iliac spine (PSIS) landmarks (see below). Such “optimal” thickness has been taken into account as the value of LLD in the subsequent statistical computations.

Between the measurement of IO and WCO, the patient was free to sit and relax for a few minutes. The full acquisition session for each subject was generally completed within 30 minutes, from the time of the subject’s arrival at the posture analysis laboratory, through the full process of assessment, to the final biomechanical report containing a complete set of IO plus WCO measures.

The full analytical and mathematical descriptions of the entire procedure is beyond the aim of this paper, wherein the main features only will be described. Full details can be found in the research papers cited below [12,35,37,48].

Before averaging, a degree of pre-processing of the acquired 3D raw data is necessary in order to comply with clinical analysis requirements. First, the subject's local co-ordinate system must first be defined; for this, we have used the general definitions provided by the Scoliosis Research Society [67]. However, in distinction to the procedure used by Stokes et al. [67], PSIS rather than ASIS landmarks have been considered in the definition of subject's local co-ordinate system, so as to reduce propagation errors and/or other interference deriving from pelvis torsion in subsequent calculation of spinal parameters.

So, in a right handed system: the frontal-coronal (YZ) plane is the vertical plane containing the PSIS (where Y axis is the horizontal medio-lateral axis belonging to this plane starting from the projection of S1 landmark on the frontal-coronal plane -defined as the origin of the subject’s local reference system- pointing from the right to the left of the body. Z is the vertical axis pointing up from origin of the system; sagittal plane is the vertical one orthogonal to the frontal-coronal plane passing through the origin of the system (X axis is orthogonal to the previous Y and Z axes pointing forward to the body defining the Anterior-Posterior direction); the transverse-horizontal plane is that orthogonal to both the previous two planes passing through the origin of the subject’s reference system (see S1 Fig).

Once having determined this individual system, a rotation is performed within each frame in order to align the subject’s co-ordinates with the global reference co-ordinates. Once the alignment is complete, it is possible to proceed to a proper averaging of all acquired frames. Based on measurements of the eleven 3D spinous processes, data are interpolated using cubic splines [68] in order to assess the position of each unlabelled spinous process and intervertebral disc. This allows us to capture anatomical structure and location with strict accuracy relative to the mathematical-geometrical representation.

After interpolation, the space-curve modelling of the spine is analytically represented by means of three parametric functions x(t), y(t), z(t) (the parameter being t > 0). A specially developed smoothing and differentiation procedure for cubic spline interpolated data is applied to these functions [12,56,57,69]. All the 2D clinical parameters claimed for the correct description and biomechanical characterisation of spinal morphology, usually calculated via a radiographic image, are derived from the 3D reconstruction. Moreover, a set of significant biomechanical variables describing the three-dimensional nature of body posture is computed. The results of filtering signal processing are used to provide the inputs of the automatic procedure for the identification of the limit-vertebrae (i.e. vertebrae marking the beginning and the end of each identified curve in both planes) defined as curve inflection points.

**Fig 4:**
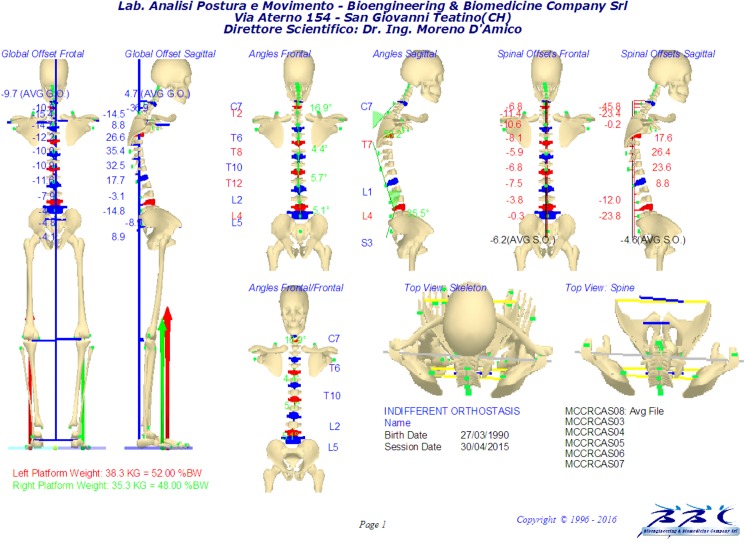
Standard graphical report an example of skeleton model reconstruction and parameters extraction.

In particular, the frontal and sagittal spine shapes are derived from the filtered 3D analytical representation of the spine by mathematical projection onto the respective planes. Subsequently frontal and sagittal spine shape curves are processed separately and the first and second derivative functions are assessed. The analysis extends from the upper labelled C7 down to the S3 spinous processes: thus, the inflection points are selected under analytical constraint, and in fact, these are the points at which the second derivative of the two average curves crosses the zero line [70]. From the values of the first derivative functions (i.e. the value of the tangents to the curve) at these inflection points, the Cobb and Kypho-Lordotic angle computation are straightforward (see S2 Fig). In this way kyphosis (i.e., backward-facing convex curve) and lordosis (i.e., forward-facing convex curve) are properly identified according to real spine curvature spatial changes at the limit-vertebrae: they are no longer restricted to specific thoracic or lumbar anatomical regions. This enables kyphosis to be identified as longer or shorter than the thoracic anatomical region, and in the same manner, lordosis can extend from the lower thoracic level to the sacral region. For this reason, the words “thoracic” and “lumbar” will be used with inverted commas in the remainder of this paper: as explained above, kyphosis and lordosis are not strictly limited to these anatomical regions. In addition, for a direct comparison with the X-Ray based Kypho-Lordotic angle values presented in the literature [71–74] the sagittal plane spine angle values have also been assessed for the most reported anatomical regions (T1—T12) as well as T4—T12 for kyphosis and T12—L5 for lordosis. After computation, a graphical report summarizes and represents the 3D full skeleton reconstruction and the related computed quantitative parameters (Fig 4).

In this paper, the total number of computed quantitative biomechanical parameters has been limited to n = 25 as shown in Table 2.

**Table 2:** List of quantitative biomechanical parameters considered by this research; and related abbreviations (n = 25).

Abbreviation	Description	Definition
ASO	**A**verage Frontal **S**pinal **O**ffsets	ASO are computed as the mean of horizontal distances of each labelled spine landmark with respect to the vertical axis passing by S3; values <0 give offsets towards the left side, >0 towards the right side.
AGO	**A**verage Frontal **G**lobal **O**ffsets	AGO are the means of the of horizontal distances of each labelled spine landmark with respect to the vertical axis passing through the mid-point between the heels; values <0 give offsets towards the left side, >0 towards the right side.
|ASO|	|**A**verage Frontal **S**pinal **O**ffset|	Absolute value disregarding the side
|AGO|	|**A**verage Frontal **G**lobal **O**ffset|	Absolute value disregarding the side
∆ASIS	∆**A**nterior **S**uperior **I**liac **S**pine	Height difference in the frontal plane as computed from ASIS levels (Right–Left Levels: sign indicates the higher side: level >0 means right higher than left and vice versa)
∆PSIS	∆**P**osterior **S**uperior **I**liac **S**pine	Height difference in frontal plane as computed from PSIS levels (Right–Left Levels: sign indicates the higher side: >0 means right higher than left and vice versa)
|∆ASIS|:	|∆**A**nterior **S**uperior **I**liac **S**pine|	Absolute ASIS height difference in frontal plane disregarding the side
|∆PSIS|:	|∆**P**osterior **S**uperior **I**liac **S**pine|	Absolute PSIS height difference in frontal plane disregarding the side
PT	**P**elvis **T**orsion = ∆(∆ASIS-∆PSIS)	values >0 indicate posterior rotation of right side with respect to the left Innominate bone while values <0 denote the opposite. Rotations are visualised around a horizontal axis running through the symphysis pubis.
|PT|	|**P**elvis **T**orsion| = ∆|(∆ASIS-∆PSIS)|	Absolute value disregarding the side
PW	Pelvis Width defined as Inter Hip Joint Centres Distance	Distance between left and right Hip Joint Centres
**CA1**	1^st^ **C**obb **A**ngle	Cobb angles of the largest “spinal deformities” found in the frontal plane
**CA2**	2^nd^ **C**obb **A**ngle	Cobb angles of the 2^nd^ largest “spinal deformities” found in the frontal plane
LLD	**L**eg **L**ength **D**iscrepancy	Right–Left Hip centres Levels: sign indicates the higher side: >0 right, <0 left) To consider both anatomical and functional LLD, the “optimal” underfoot wedge correction thickness has been considered as the value of LLD (see text)
|LLD|	|**L**eg **L**ength **D**iscrepancy|	Absolute value disregarding the side
∆UL	∆**U**nderfoot **L**oad	Left vs. Right sides Body Weight Percentage Difference
TKA	“**T**horacic” **K**yphosis **A**ngles	The computed angle in the identified kyphosis: see text for definition and explanation.
LLA	“**L**umbar” **L**ordosis **A**ngles	The computed angle in the identified lordosis: see text for definition and explanation.
KCL	**K**yphosis **C**urve **L**engths	As explained in the text kyphosis and lordosis curves are not strictly thoracic or lumbar, and they can vary in location and length. The length is computed in terms of the number of vertebrae between the identified limit vertebrae.
**LCL**	**L**ordosis **C**urve **L**engths	
KLV	**K**yphosis **L**imit **V**ertebrae occurrence along the spine	As explained in the text kyphosis and lordosis can vary in location and length along the spine. So for each spinous process, it is possible to count the number of times it is identified as a limit vertebra for kyphosis and lordosis among all subjects in the sample. The occurrence of KLV and LLV is expressed as a percentage; e.g. C7 = 10% in the “thoracic” kyphosis of Female group means that 10% of female subjects presented C7 as upper limit vertebra in the “thoracic” kyphosis.
**LLV**	**L**ordosis **L**imit **V**ertebrae occurrence along the spine	
T1-T12	Fixed Thoracic Kyphosis Angles	The X-ray literature consistently reports kyphosis as bound by the thoracic region (see text).
T4-T12	Fixed Thoracic Kyphosis Angles	The X-ray literature consistently reports kyphosis as bound by the thoracic region (see text).
T12-L5	Fixed Lumbar Lordosis Angles	The X-ray literature consistently reports lordosis as bound by the lumbar region (see text).

### Statistical analysis

All statistical analyses were carried out using the Real Statistics Resource Pack software (Release 4.7) [75] with a significance level of α < 0.05. Due to the interval nature of the outcome variables, tests for normality were carried out (Shapiro, Wilk), followed by tests for homogeneity of variance (Fisher test) where applicable. The statistical analysis has been applied and managed at various levels beginning with general descriptive statistics for the entire sample.

#### Group statistical analysis

The sample has been subdivided into two groups, Males and Females. Postural characteristics for both genders were compared for each variable of interest, applying a set of unpaired t-Tests (or the Mann-Whitney non-parametric equivalent tests (where assumptions for normality and/or homogeneity of variances were not satisfied) [75,76]. The Males vs. Females assessment was carried out by analysing both the IO and the WCO separately. After investigating differences between the sexes, we proceeded to a second statistical analysis block, seeking differences in IO vs. WCO. This was accomplished by mean of paired t-Tests (or Wilcoxon signed-rank non-parametric equivalent tests when normality assumptions were not satisfied) [75,76]. In this case, the sub-group of 100 subjects (out of the full sample of 124) for which WCO measures were available was examined. In performing the IO vs. WCO test, the outcomes of the previous Males vs. Females comparison were considered. For those variables that presented a statistically significant difference between Males and Females, comparisons were made between genders as separate groups. Conversely, for those variables without a statistically significant difference between sexes, the comparison was performed without distinction between the Male and Female sub-groups.

#### Intra-subject statistical analysis

Extending the statistical analysis to the intra-subject level, we further investigated the IO vs. WCO comparison by examining how the application of underfoot wedge correction modified subject posture in terms of improving, worsening or having no effect (*Unchanged*) on the original upright standing posture. To this end, we selected ten main variables of interest (out of the total n = 25 considered for group statistics). These variables were grouped by their specific characteristics into frontal and sagittal plane parameters plus an additional parameter describing load balancing (see Table 3).

**Table 3:** List of parameters considered for IO vs. WCO intra-subject comparison and summarizing indexes.

Global Summarizing Index	Parameters		Descriptions	Specific Summarizing Indexes
GPI Global Postural Index	**|ASO|; |AGO|; CA1; CA2; |∆ASIS|; |∆PSIS|**	N. 6 parameters describing postural characteristics in the frontal plane		**FPI** Frontal Postural Index
	**TKA, LLA, |PT|**	N. 3 parameters describing postural characteristics in the sagittal plane		**SPI** Sagittal Postural Index
	**∆UL**	N. 1 parameter describing conditions of load balancing/unbalancing i.e. underfoot loading patterns, in terms of left vs. right sides body weight percentage difference		**LBI** Load Balancing Index

For each of such variables, a t-Test has been performed between the values measured per each subject in the IO and in the WCO postures. When no statistically significant difference is found, the actual postural parameter has been classified *Unchanged*. When the value of a selected parameter showed a statistically significant change we defined it as an Improvement in the following situations:

- Frontal parameters: theoretically the spine should be straight and left and right sides symmetrical. Given the fact that frontal postural parameters quantify how symmetrical is the subject’s upright standing posture as well as the presence and value of eventual spinal curves we assumed that the optimal theoretical value had to be zero for any considered frontal parameters. So, when the changes approached this value, it was defined as an *Improvement*.
- Sagittal plane: in this case, there are no theoretical optimal reference values, with the exception of |PT| where zero would indicate a perfectly symmetrical pelvis. A wide range of normative values has been proposed in the literature both for thoracic kyphosis (20° to 50°) and lumbar lordosis (20° to 70°) computed via X-ray [51]. As we measured the sagittal profiles of spinous processes labelled by skin markers, we decided to consider as reference values the mean profile values calculated from our sample population. Specifically, we defined kyphosis and lordosis as backward- and forward-facing convex curves (with related angle values) without considering their specific location along the spine. When statistically significant changes approached this mean value, it was classified as an *Improvement*.
- ∆UL: the optimal theoretical condition is achieved when there is a perfect balance of underfoot load distribution between left and right sides; therefore, we concluded that there was *Improvement* when changes approached this condition.

*Worsening*: each time a statistically significant change differed from the definitions of Improvement (as above) it was concluded that *Worsening* had occurred.

### Summarizing indexes

Given the fact that such comparisons provided a mix of *Improvements* and *Worsening* per each subject, four summary indexes were devised for each subject in order to synthetically define the effect of induced underfoot wedge correction. When an *Improvement* is detected, a +1 score is associated to the related variable; if a *Worsening* is revealed, the related score is -1; if no statistical change is found, the score for this variable is zero. In this way, we devised a “Global Postural Index” (GPI_i,_ see Table 3), which is simply the sum of scores obtained for all variables for the i^th^ subject. In the same way, we built up the Frontal Plane Index (FPI_i_), the Sagittal Plane Index (SPI_i_) and the Load Balancing Index (LBI_i_) each one defined by the sum of scores for the variables of the related group.

A positive GPl_i_ indicates a global *improvement* for the subject resulting from the transition from IO to the WCO posture; conversely, a negative index indicates a global *worsening*. Finally, a GPl_i_ equal to zero reveals that no significant global changes can be detected between the two postural conditions; or perhaps that the number of improved parameters equals the number of worsened parameters. These conclusions have an equally straightforward application to FPI_i_, SPI_i_, LBI_i_.

In order to simplify the readability of each index value for the whole population, they are expressed in percentages of the total. That is, the sum of scores obtained for each subject allows us to calculate the population as GPI_P_ = Σ_i_GPI_i_/N: i.e., the number of Improvements, *Worsening* and *Unchanged* with respect to the whole sample size N. Similarly FPI_P_ = Σ_i_FPI_i_/N_,_ SPI_P_ = Σ_i_ SPI_i_/N and LBI_P_ = Σ_i_ LBI_i_/N scores provide detail for the analysis of specific planes or variables.

### FPI vs. LBI statistical relationship

We also investigated the presence or absence of a possible statistical relationship between changes in the frontal plane behaviour and changes in the load balancing/unbalancing, the latter being mostly connected to frontal plane variations. To do this, we devised a contingency table in which we crossed FPI and LBI *Improvements*, *Worsening* or *Unchanged* respectively. This resulted in a Chi-Square Test of Independence.

### Influences on loading patterns

We further investigated factors which could influence ∆UL. To this aim, we performed a 2 Ways ANOVA Test (α = 5%) using sex and loading patterns as factors. In this case, the investigation refers to the question of whether ∆UL is different between genders, between those who charge the higher load (in terms of body weight percentage) on the shorter or longer leg or finally whether there is a relationship between gender and pattern of charging. This test was carried out both in IO and in WCO separately.

### Independence test

Finally, as our concluding evaluation, we applied a Chi-Square Test of Independence to the entire sample, to verify whether specific pairs of found conditions were statistically reciprocally independent. In particular, we studied the following:

1. the independence/dependence relationship between the side on which the shorter or longer leg is located, and three elements: gender, loading pattern and pelvis torsion (PT);
2. whether, when LLD is present, the subjects tended to shift a greater load onto the shorter or the longer leg;
3. the independence/dependence relationship between gender and pelvis torsion (PT) taking into account whether the anterior or posterior rotation occurs on the shorter or longer leg;
4. the independence/dependence relationship between loading patterns and PT.

## Results

### Group statistical analysis

The Male vs. Female comparisons showed only a few statistically significant differences (Table 4). Thereby, females showed a significantly shorter PW (p<0.01) and a lower value for |PT| in the WCO condition (p<0.05). Despite the fact that males were taller than females, LLD, as well as the effective length of the shorter leg, showed no statistically significant gender-related differences. Overall, the right leg was the shorter one in 48 subjects and the left in 52 subjects.

**Table 4:** The results of Male vs. Female comparison of 3D posture parameters: Only the parameters that showed statistically significant differences are reported.

	Males vs. Females comparisons			
3D POSTURE PARAMETERS	Unpaired MALE vs. FEMALE: INDIFFERENT Orthostasis		Unpaired MALE vs. FEMALE: WEDGE Corrected Orthostasis	
	Significance level	Cohen Effect Size	Significance level	Cohen Effect Size
**|PT|** (Mann-Withney Test)	NS	0.028	P<0.05	0.430
**PW** (t-Test)	P<0.01	0.537		
**LLA** (t-Test)	P<0.001	1.276	P<0.001	1.104
**T12-L5** (t-Test)	P<0.001	1.500	P<0.001	1.385
**LCL (n. of Vertebrae)** (Mann-Withney Test)	P<0.001	0.781	P<0.001	1.096

Females display “Lumbar” lordosis with a higher angle value and at the same time a shorter curve length (in terms of the number of included vertebrae) as compared to Males both in IO and in WCO. The same results are obtained for the fixed bounded T12-L5 Lumbar anatomical region.

**Fig 5:**
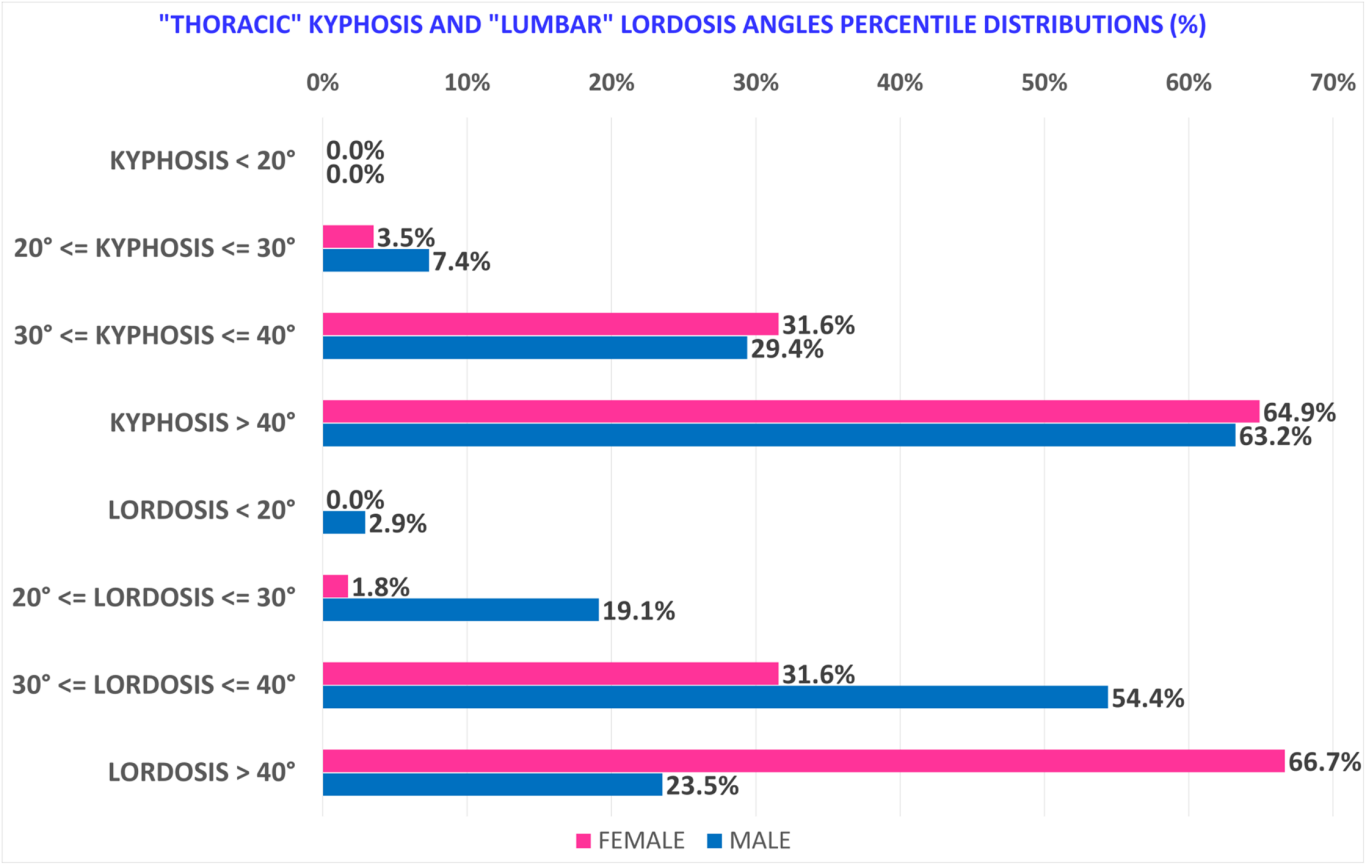
Male and Female “Thoracic” kyphosis and “Lumbar” lordosis angle values (deg.) as a percentile distribution.

**Fig 6:**
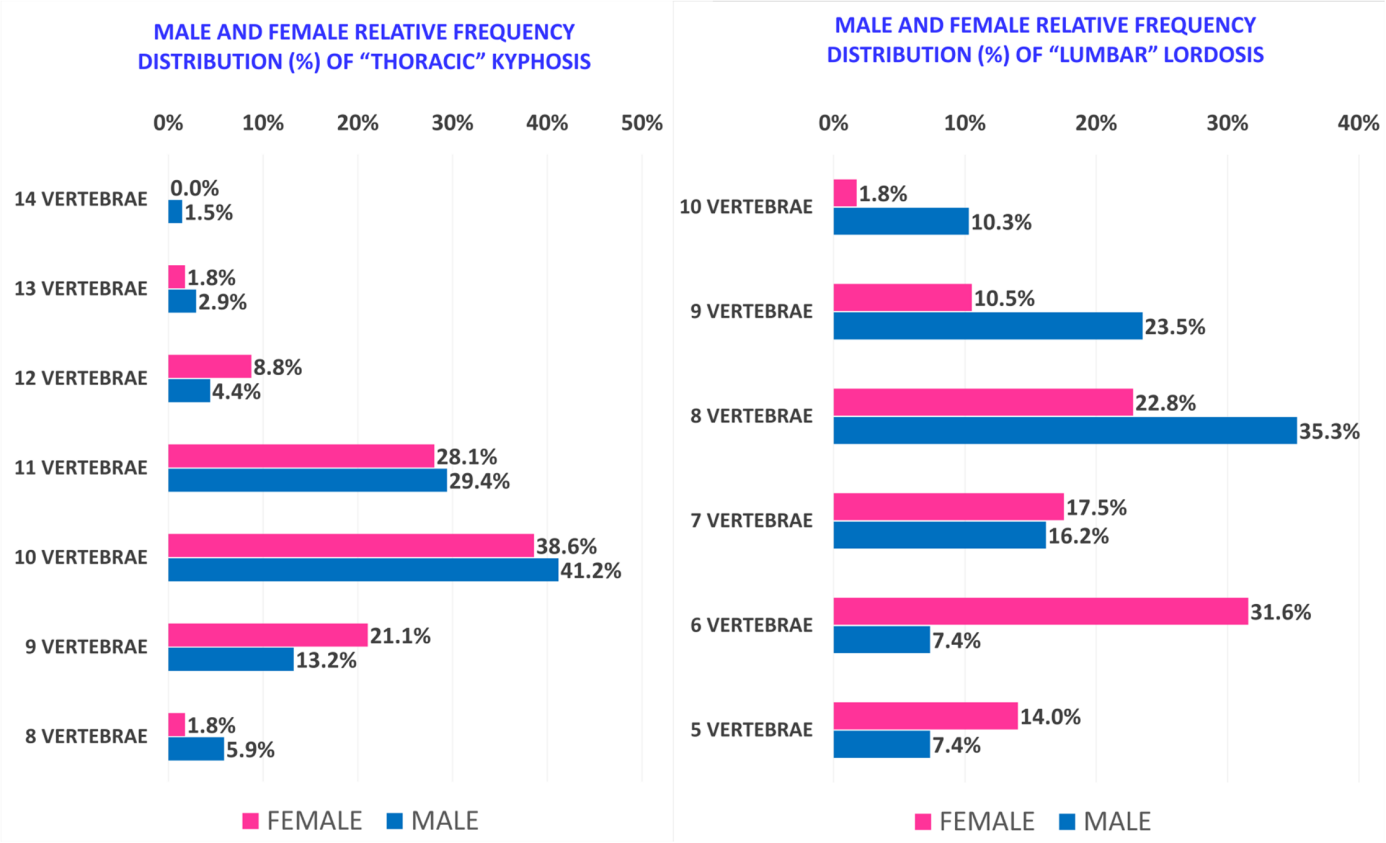
Male and Female relative frequency distribution (percentage related to the total) of “Thoracic” kyphosis and “Lumbar” lordosis curve lengths by the number of included vertebrae.

**Fig 7:**
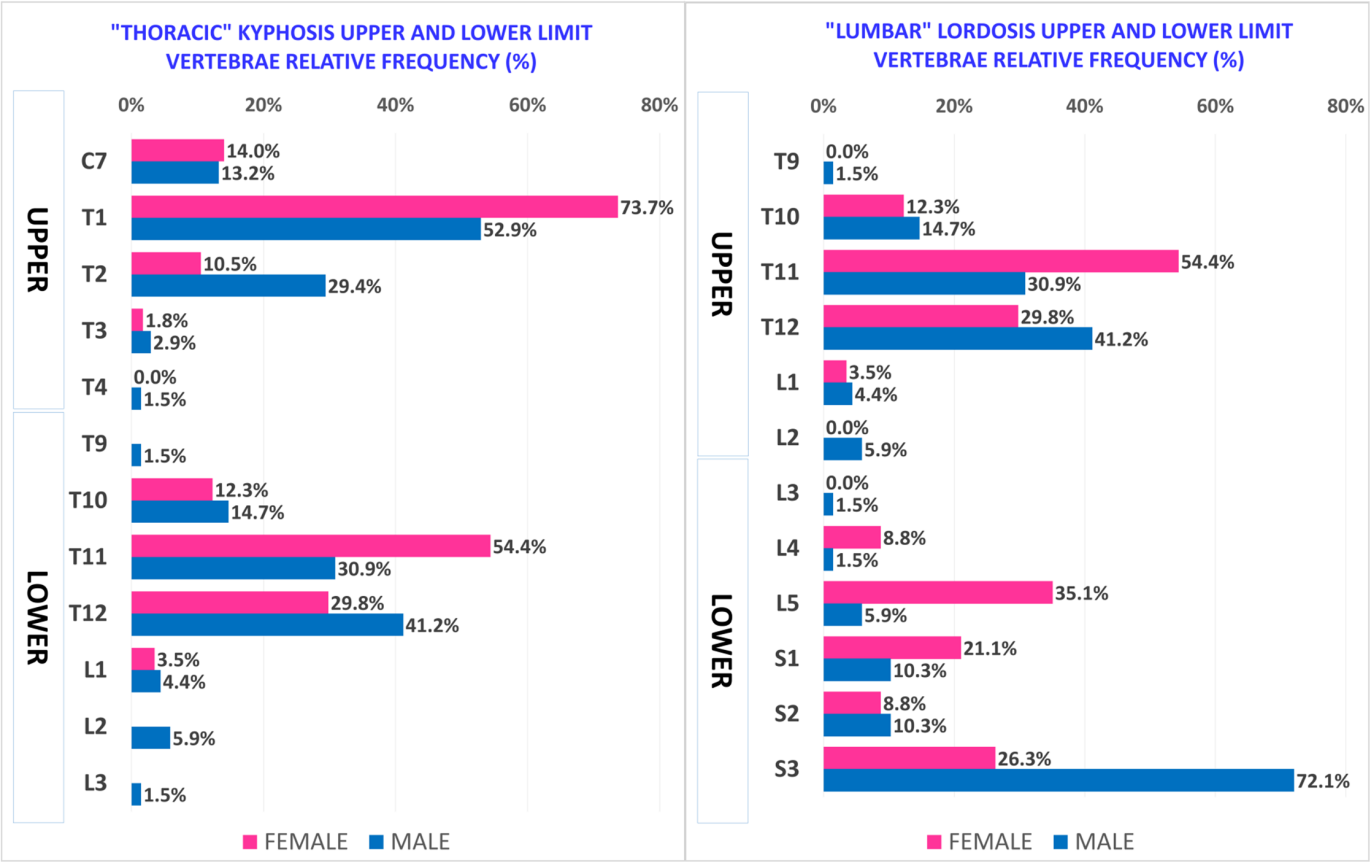
Relative frequency of Male and Female “Thoracic” kyphosis and “Lumbar” lordosis Upper and Lower limit vertebrae (KLV, LLV: see Table 2 for definitions) along the spine. The frequency of KLV and LLV is expressed by each identified limit vertebrae shown as a percentage of the total number in the sample.

“Thoracic” kyphosis, conversely, is similar in both sexes as regards angle value and curve length, and in both IO and WCO. In addition to statistical tests, we also identified and described the distributions of “Thoracic” kyphosis and “Lumbar” lordosis angle values (Fig 5), curve lengths (Fig 6) and Upper and Lower limit vertebrae occurrence rate (Fig 7). The results showed that about two thirds of Females present a “Lumbar” lordosis angle value higher than 40° (Fig 5) and more than 70% present a “Lumbar” lordosis length of between 6 and 8 vertebrae, with the peak frequency being 31.6% with lordosis length of 6 vertebrae (Fig 6).

In contrast, more than 75% of Males have a curve angle value below 40° (Fig 5) and 75% also have a “Lumbar” lordosis length between 7 and 9 vertebrae, with the peak frequency being 35.3% with lordosis length of 8 vertebrae (Fig 6). Furthermore, taking into account the occurrence rate of Upper and Lower limit vertebrae, Females as compared to Males tended to have kyphosis and lordosis curves at a higher position in the spine (Fig 7). “Thoracic” kyphosis was found to be identical in Males and Females not only in the average values of angle and curve length but also in distribution (angle values as a percentile are substantially superimposed) (Fig 7).

As a result of the IO vs. WCO comparisons, reported in Table 5, a general improvement can be observed in all the parameters that quantify balance and underfoot load distribution in the frontal plane. In addition, pelvis obliquity is reduced. For Males a slight, though statistically significant, increase is registered in pelvis torsion (irrespective of which side anterior or posterior rotation occurs), in “Lumbar” lordosis angle value (LLA) and in fixed bounded (T12-L5) Lumbar anatomical region angle value.

**Table 5:** The Results of IO vs. WCO comparison of 3D posture parameters.

Parameter	Significance level	Cohen Effect Size
**ASO** (t-Test)	P<0.01	0.309
**AGO** (Wilcoxon Test)	P<0.001	0.624
**|ASO|** (Wilcoxon Test)	P<0.001	0.672
**|AGO|** (Wilcoxon Test)	P<0.001	1.144
**|∆ASIS|** (Wilcoxon Test)	P<0.01	0.322
**|∆PSIS|** (Wilcoxon Test)	P<0.001	1.683
**|PT| Male**(Wilcoxon Test)	P<0.05	0.311
**∆UL** (Wilcoxon Test)	P<0.001	0.356
**LLA Male** (t-Test)	P<0.05	0.314
**T12-L5 Male** (t-Test)	P<0.05	0.320

The assessed Normative Data for the sample population are reported in Table 6. As regards IO, all 124 subjects within the cohort were examined and measured. However, as regards WCO, the sample size was reduced to 100 subjects (see above). Those variables that produced a statistically significant difference between Male and Female groups are listed separately, by gender. Conversely, for variables that did not produce statistically significant differences between sexes, the reported value was obtained by combining all Males and Females into a single sample. In this way, a total of thirty 3D quantitative postural, spine morphology and pelvic parameters were obtained.

**Table 6:** Normative data (3D postural parameters) for IO and WCO.

3D Posture Parameters	Indifferent Orthostasis (n = 124)		Wedge Corrected Orthostasis (n = 100)	
	Mean± SD	Ranges: min-max	Mean± SD	Ranges: min-max
**ASO [mm]**	-3.71 **±** 7.02	-21.9–17.7	-2.33**±**4.20	-14.5–9.0
**AGO [mm]**	-8.30 **±** 11.89	-40.3–27.9	-2.29**±**3.58	-13.0–4.7
**|ASO| [mm]**	6.31 **±** 4.81	0.1–21.9	3.82**±**2.89	0.2–14.5
**|AGO| [mm]**	11.85**±** 8.32	0.0–40.3	3.04**±**2.97	0.0–13.0
**CA1 [degree]**	10.8° ± 5.18°	1.5°–26.1°	10.50±5.11	1.4°–31.6°
**CA2 [degree]**	7.28° ± 4.16°	0°–16.8°	6.8±4.14	0°–22.6°
**∆ASIS [mm]**	0.53 ± 9.45	-21.0–29.9	-0.70±7.82	-26.9–23.8
**∆PSIS [mm]**	0.26±5.49	-11.0–10.2	-0.18±1.77	-5.6–3.8
**|∆ASIS| [mm]**	7.76±5.37	0.0–29.9	5.75±5.31	0.0–26.9
**|∆PSIS| [mm]**	4.94±2.37	0.3–11.0	1.36±1.13	0.0–5.6
**|PT| Full [mm]**	5.65±4.24	0.0–21.6	6.06±5.22	0.1–30.5
**|PT| Male [mm]**	5.33±4.48	0.0–21.6	6.92±6.22	0.3–25.2
**|PT| Female [mm]**	5.45±3.92	0.1–14.9	4.65±4.11	0.1–15.8
**PT Full [mm]**	0.20±7.08	-18.7–21.6	-2.93±7.46	-30.5–14.9
**PT Male [mm]**	0.18±6.99	-13.8–21.6	-0.36±9.35	-25.2–23.7
**PT Female [mm]**	0.38±6.74	-14.9–12.9	-0.70±6.2	-15.8–14.5
**LLD [mm]**	9.37±3.31	6.0–23.0	—	—
**∆UL (%BW)**	1.9 ±6.13	-16.4–17.8	-0.58±5.26	-10.2–21.1
**PW (IHJCD) Male [mm]**	167.05±15.3	132.5–212.2		-
**PW (IHJCD) Female [mm]**	158.81±14.8	125.3–201.7	—	—
**TKA [degree]**	45.98°±8.75°	25.2°–76.3°	46.46° ± 8.39°	21.8°–65.1°
**LLA Males [degree]**	32.8±8.09	12.5°–54.8°	34.19° ± 9.16°	14.4°–62.0°
**LLA Females [degree]**	44.20±9.66	24.3°–68.3°	44.49° ± 9.24°	25.2°–68.5°
**T4-T12 [degree]**	31.9±7.65	14.2°–51.6°	31.8° ± 7.48°	8.1°–53.4°
**T1-T12 [degree]**	42.56±8.89	15.0°–64.8°	42.92° ± 8.94°	18.3°–63.7°
**T12-L5 Males [degree]**	25.65°±9.38°	4.0°–53.0°	26.9° ± 9.2°	8.7°–46.8°
**T12-L5 Females [degree]**	39.45±8.97	16.9°–60.0°	39.70° ± 9.27°	17.0°–59.2°
**KCL (n. of Vertebrae)**	10.25±1.11	7–14	10.23 ±1.06	7–13
**LCL Males (n. of Vertebrae)**	7.9±1.34	5–10	8.12±1.21	6–10
**LCL Females (n. of Vertebrae)**	6.84±1.36	4–10	6.67±1.43	4–10

## Intra-subject statistical analysis

Regarding the Intra-Subject Statistical Analysis (Table 7), some general results can be described. For each considered quantitative parameter, the number of obtained *Improvements*, *Worsening*, *Unchanged*, is reported as percentages of the total population. In the frontal plane, |∆ASIS| and |∆PSIS|, |ASO| and |AGO| all present a substantial and significant improvement. On the other hand, CA1 appears to be relatively constant: i.e., 2/3 of the sample is classified as “*Unchanged*”; and while CA2 showed slightly more change, with 30% showing “*Improvement*”, 52% nonetheless also remain classified as “*Unchanged*”. Taking into account the summarizing indexes, the FPI depicts a substantial frontal plane postural *Improvement* (86%) after the transition from IO to WCO.

**Table 7:** The results of intra-subject statistical analysis.

Quantitative Parameter* and Summarizing Indexes*	IMPROVEMENTS (%)	WORSENINGS (%)	UNCHANGED (%)
**|ASO|**	69%	20%	11%
**|AGO|**	79%	3%	18%
**CA1**	17%	16%	67%
**CA2**	30%	18%	52%
**|∆ASIS|**	64%	36%	0%
**|∆PSIS|**	93%	7%	0%
**|PT|**	46%	49%	5%
**TKA**	13%	7%	80%
**LLA**	17%	9%	74%
**FPI**	86%	5%	9%
**SPI**	43%	40%	17%
**LBI / (∆UL)**	37%	19%	44%
**GPI**	82%	9%	9%

* *To simplify the readability of each reported value they are expressed in percentages of the total (see text)*

In the sagittal plane, Pelvis Torsion (|PT|) showed a significant variation: 46% of subjects showed *Improvement*, 49% *Worsenings*). Apart from this, spine angles were unchanged for a large percentage of the sample: there was no change in angle values of “Thoracic” kyphosis (TKA) for 80% of subjects, or of “Lumbar” lordosis for 74% of subjects. In addition, the composition of scores in the summarizing sagittal parameter index (SPI) shows 20% of the sample was classified as “*Unchanged*” while the remainder was almost equally distributed between “*Improvement*” and “*Worsening*”.

The results for the LBI parameter indicate that almost half of the sample (44%) did not change their underfoot loading pattern when the wedge correction was applied, while a 37% (*Improvement*) tended towards a re-balancing of loads.

### FPI vs. LBI statistical relationship

The Chi-Square Test of Independence, cross-testing the FPI and LBI indexes established that the outcomes are reciprocally independent in terms of *Improvement*, *Worsening*, or *Unchanged*.

### Influences on loading patterns

The 2 Ways ANOVA Test was applied in relation to the ∆UL, in IO, for the following four groups:

1. Shorter Leg Loaders–Male
2. Longer Leg Loaders–Male
3. Shorter Leg Loaders–Female
4. Longer Leg Loaders–Female.

The results of this test showed no significance either in the main effects or in the interaction term; therefore, no statistical differences are to be found among mean ∆ULs for the four groups.

From this, we proceeded to a **Data Pooling** and subsequent **2 Single Factor ANOVA Test** (α = 5%). The comparison of Males vs. Females confirmed that there were no differences in ∆ULs. Conversely, by subdividing the whole sample into two groups, of longer and shorter leg loaders, the latter group presented a statistically higher ∆UL value, in relation to the longer leg loaders (p<0.05; μ_LL_ = 4.0%, μ_SL_ = 5.8%). Interestingly, this differential result disappeared in the WCO posture (i.e., there was no difference in ∆UL value between shorter vs. longer leg loaders); this result further highlighted the beneficial effect of underfoot wedge correction as a method of rebalancing and/or equalisation of LLD. This additional useful effect in reducing differences between shorter vs. longer leg loaders completes the range of improvements already found in the Intra-subject assessment.

### Independence test

Finally, we applied the Chi-Square Test of Independence in IO, as regards the following factors: the side on which the longer/shorter leg is found; gender; loading patterns; and pelvis torsion (PT). Our conclusions are set out as follows.

There is no dependence between the side on which the longer/shorter leg is found and any of the other factors: gender, loading patterns, or pelvis torsion (PT). In other words, the shorter or longer leg can be either the right or the left, independent of gender, loading pattern and/or the PT. When an LLD is present, Females more frequently place a higher load on the shorter leg (χ^2^ p-value = 0.0084). Even where the same tendency was found in the Male group, the Chi square test did not reach the level of significance. We, therefore, concluded that there is no difference in loading pattern between genders.

We also concluded that the probability that a subject presents a posterior rotation of Innominate bone on the side with the shorter or longer leg is independent of his/her sex. Conversely, by checking the Male and Female groups separately, we found statistical evidence that Females more frequently present anterior rotation of Innominate bone on the shorter leg side (χ2 p-value = 0.024). Within the Male group, this Chi square test does not reach the level of significance.

In contrast, we concluded that the probability that a subject presents a posterior/ anterior rotation of Innominate bone on the shorter or longer leg side is, in fact, dependent on his/her loading pattern: i.e., it does depend on whether he/she is a shorter or longer leg loader (χ2 p-value = 0.028). We found that, as regards the ipsilateral side, the longer leg loaders have a significantly higher probability of posterior rotation; while the shorter leg loaders have a significantly higher probability of anterior rotation.

## Discussion

The current study is an observational cross-sectional study. It yields Normative Data i.e. the physiological standard for a total of 30 selected 3D quantitative postural, spine morphology and pelvic parameters that in combination describe the full-skeleton upright neutral standing attitude we define as Indifferent Orthostasis (IO). This data set constitutes a baseline reference system for the study of any kind of postural disorder and posture-related orthopaedic and neurological pathology. In addition, being very widely applicable, it can be used beneficially for the study of postural characteristics connected to different sports activities as well as to evaluate postural change during growth and/or ageing processes. The researchers aim to continuously expand this research, over time, increasing the sample population to strengthen the reliability of this database.

The 3D opto-electronic stereo-photogrammetric approach methodology has been preferred in building up this database of accurate, normative data because it allows us to overcome, as described in the introduction, the constraints [1] that still affect either manual and “single shot” radiological methods or other non-invasive non-ionizing instrumental techniques previously developed to address the potential harmfulness of X-Ray, but that present strong limitations all of which limit their use mainly to follow-up monitoring. Among the various 3D opto-electronic stereo-photogrammetric original approaches presented in the literature [12,13,24–39,41], we focus, in this paper, on the special protocol and elaboration technique formerly presented in D’Amico et al. 1995 [12]. This protocol allows users to obtain a complete 3D parametric biomechanical model of the human skeleton, with particular detail in the 3D capture and reproduction of spine shape, thus providing an accurate and complete representation of the body’s standing posture. This measurement system contains three main sources of potential error: the accuracy of the 3D stereo-photogrammetric capture; operator-dependent skin marker positioning; and data processing. In the first instance, present-day high-resolution stereo-photogrammetric systems provide very high 3D accuracy in 3D marker position reconstruction (0.3–0.4mm range on a 3mx3mx2m working volume in GOALS system for the present study) using well-established and easy-to-manage mature stereo-photogrammetric procedure [64,65]. As regards the second source of potential error, prior studies [22,31,77–81] have demonstrated via X-Ray and Magnetic Resonance Imaging that skilled operators are able to perform accurate positioning by palpation (with a relatively low intra- and inter-examiner error with respect to subject postural adjustment variability). Third, data processing could be a primary source of data corruption if improper signal processing techniques are used. This is particularly true when 3D spine shape and its related 1st and 2nd derivatives (necessary to proceed to an assessment of curve angles) must be identified using only 11 points [12,56,57]. Prior work by D’Amico et al. 1995 [12] originally introduced an efficient and specially devised numerical processing technique, showing the maximal error of less than 1° (approximately) for the Cobb computed angle on a curve of about 65°, modelled with a simulated sinusoidal data series with superimposed white noise (σ = 1mm). A subsequent study on scoliotic patients [39] presented an *in vivo* comparison between frontal plane Cobb angles computed using stereo-photogrammetry and AP (anterior-posterior) X-Ray images. Results showed that Cobb angles measured via X-Ray matched those assessed via a stereo-photogrammetric system.

Upon noticing that, of the first 24 analysed subjects, all presented with some amount of leg length discrepancy (LLD) it was decided to extend the study, to investigate the influence on posture (if any) that might be produced by promoting equalisation between the lower limbs. This equalisation was achieved by placing a suitable corrective wedge under the foot in order to reduce or eliminate LLD. Thus, a further neutral standing posture measurement was conducted and analysed for the remaining 100 subjects in the sample; we called this “Wedge-Corrected Orthostasis” (WCO). Postural and spine morphology data were compared between genders. Where statistically significant differences were found, the Normative Data were subdivided according to gender. After all measurements had been taken, some degree of LLD was found in 100% of analysed subjects. LLD is reported as relatively common, affecting up to 90% of the population, with an average value of 5.2±4.1mm: Figures established by Knutson [66] after pooling data from a number of studies on anatomic LLD, evaluated with precise radiographic methods of millimetric accuracy. Our evaluation takes into account both anatomic and functional LLD. This could explain why both the percentage and the average LLD value found in this study produced a higher result (9.37±3.31mm quantified via the thickness of the “optimal” wedge correction). The magnitude of the LLD results was independent of gender, notwithstanding Males were statistically taller than Females. This is in agreement with a number of reported studies about LLD [82–85]. In our analysed sample, there was no evidence of shorter leg length being more prevalent on one side of the body (right leg shorter: 48%, left leg shorter: 52%) as distinct from other studies where the right leg was reported to be anatomically shorter more often [66]. Conversely, in our analysis, the equivalence of the magnitude of the discrepancy between sides was found to be in full agreement with reports by various authors in the literature [66].

Only five statistically significant differences between genders have been found: Pelvis Width (given by IHJCD), “Lumbar” Lordosis angle value, Lumbar Curve Length, T12-L5 anatomically-bound Lumbar region angle value and the |PT| parameter. The |PT| parameter changes in Males but not in Females when transitioning from IO to WCO even if it shows no difference between sexes in IO: evidence therefore of significant magnitude discrepancy in WCO.

Pelvis width was found to be greater in Males than in Females. This is consistent with Seidel et al. 1995 [50] who found, on cadaver specimens, female pelvises significantly and proportionally smaller than male pelvises. Interestingly this characteristic could be connected with the different outcomes between genders in relation to |PT| changes when the wedge correction was applied (above).

The results showed that “Lumbar” lordosis angle value was greater in Females than in Males. This finding is in full agreement with a number of studies using X-Ray based measurement [71–73,86–89], as is also the found absence of gender influence in “Thoracic” kyphosis. Similarly, in a study using a 3D electromechanical digitizer to derive curvature angles for the region of the spine between T12-L1 and S2, Norton et al. [90] found that the lordosis angle (calculated using the ratio between lordosis depth and length) was 13.2° larger for women than for men. On the other hand, a study conducted using the Cobb angle measurement on 2D radiographs of an older adult Greek population (99 subjects, asymptomatic, average age 52.7 ± 15 years) confirmed our findings of gender independence on thoracic kyphosis but rejected the view that lumbar lordosis (in both T12-S1 and L1-L5 regions) is sex-related [91]. A more recent study [92] conducted on 60 young healthy males and females (average age 27) demonstrated that the female spine is definitely different from the male spine but failed to reveal a significant difference between males and females in thoracic kyphosis and lumbar lordosis; this study relied upon innovative low-dose digital biplanar x-rays and three-dimensional upright analysis. “Lumbar” lordosis length, in term of included vertebrae, is shorter in Females than in Males. This finding introduces a new and important issue. The use of Cobb angle values alone does not give a complete sense of the magnitude of the curve and may be misleading if curve length and curvature is not accurately observed and quantified [51,93]. Conversely, the approach presented in this paper enables users to highlight, in a mathematically defined way, individual and group differences not only in values but also in length and position of the found curves. These important aspects have been taken into account in some X-Ray studies as well: Vialle et al. 2005 [71] noted the high variability of the position of the inflection point between kyphosis and lordosis as also determining substantial variability in the assessed angular values; while Roussouly et al. assembled all these characteristics to build up a classification system for variations in sagittal alignment in the standing position, for both normal [94] and pathological populations [5].

Few papers in the literature are focused on spine balance in the frontal-coronal plane, while a high number are focused on sagittal spine balance. Normative data from X-Ray measurements for Thoracic Kyphosis span a range of 20°-70° thus presenting a high variability among the studies [51,71–74,86,87,89,91,94–97]. In one of the earliest studies on sagittal alignment, Stagnara et al. [95] concluded that the “span of possible values of maximum kyphosis and lordosis in subjects with no spinal disease is considerable. It is therefore unreasonable to speak of normal kyphotic or lordotic curves.” This very conclusion led us to choose average sagittal plane spine morphology and balance characteristics as the basis for our intra-subject IO vs. WCO comparison, thus enabling us to delineate reasonable criteria for the definition of improvement or worsening of posture in the sagittal plane.

In any case part of the variability reported in literature, for both Thoracic kyphosis and Lumbar lordosis, depends on measurement methods, on limit vertebrae used to bound the kyphotic-lordotic regions and, last but not least, on patient positioning (especially in relation to upper arm position, to avoid occlusion of thoracic spine visibility) [98]. Despite this variability, several subsequent studies have demonstrated that sagittal alignment can be accurately measured and that these measurements are repeatable when the same subject is examined at two different points in time [97,99].

There exists a high degree of agreement between our research and the literature on X-ray-based normal sagittal spine morphology (a short review is found in S1 Table [71–74,87,89,91,94,96,100–103]) regarding the mean angle values obtained in our population by measuring the profile of spinous processes in a neutral standing upright posture with arms at the side. Due to changes in spinal profile induced by ageing [5] the data reported in S1 Table refer to populations with an age range similar to that analysed in the present study. There is particularly strong accordance with some recently published studies in which X-rays were taken in a standing position with the hands supported and shoulders slightly flexed at 45°, relatively close to the value of 30°; Marks et al. 2009 [98] established this as being comparable to measurements taken in a functional standing position with arms at the side—as in the present study. By looking at the comparison among segments T1-T12, T4-T12, L1-L5, L1-S1, further conclusions can be drawn regarding the effect of vertebrae and discs wedging. At the thoracic level, the latter seems not to be significant for a younger population, leading to a favourable geometric relationship between the shape of profile given by spinous processes and the related vertebral body shape, producing a minimal difference between dorsal surface and vertebral column curvatures. Conversely, the differences found for the magnitude of lordosis angle value when the S1 vertebra plate is taken as the lower limit might be due primarily to the relevant effect caused by the wedging of the disc at S1-L5 as reported in the literature [71,72,89]. Remarkably, Damasceno et al. 2006 [89] noticed that caudal elements of curvature, intervertebral discs L4-L5 and L5-S1 and the vertebral body L5 accounted for nearly 60% of the angular measurement of lumbosacral curvature. In fact, it is important to remark that, when for instance even just the L5-S1 wedge disc contribution (assessed to be around 15° [71,72,89]) is removed, the values we obtained both for Males and Females are seen to be in very close agreement with those found in X-ray measurement.

As established by IO vs. WCO tests, to be asymptomatic does not mean to have an optimal posture. It seems that asymmetry (associated with unbalanced postural and underfoot loads, spinal curvature in the frontal plane, and PT) is standard in both sexes. Even the LLD mean value is relatively equivalent between genders as previously reported [66], notwithstanding statistically significant differences in body height (Table 1); and approximately 82% of the sample showed improvement in the standing posture as a result of an LLD-equalising intervention. By analysing the FPI, SPI and LBI summary indexes it is evident that improvements were mainly in the frontal plane; while in the sagittal plane, more than 2/3 of the sample demonstrated no change in spine angles; the exception being pelvis torsion which showed a significant variation (46% Improvement, 49% Worsening). Some parameters showed an immediate improved response: AGO, ASO, ∆ASIS and ∆PSIS; while others showed a tendency towards improvement but with slower actualisation. For example, by looking at LBI we see that more than 1/3 (37%) of the sample showed improvement, but a greater percentage (44%) showed no change: for this parameter, it could be argued that the motor control system might possibly require a longer period of adaptation with respect to the given 2-minute interval between the two measured standing postures. This could explain the results of the Chi-Square Test of Independence, which showed that *Improvement*, *Worsening* or *Unchanged* in FPI are independent from those in LBI. The side of the body associated with the shorter/longer leg, the loading pattern and the kind of PT are all independent from gender; and additionally, there was no difference between the left and right sides. Conversely, where an LLD was present, the entire sample showed a tendency to put the higher load on the shorter leg and to more frequently present an anterior rotation of the Innominate bone on the shorter leg side. Both of these tendencies are statistically significant in Females.

Finally, there exists a relationship between pelvis torsion and underfoot loading patterns: there is a higher probability that longer-leg loaders will demonstrate posterior rotation on the ipsilateral side, as compared to shorter-leg loaders who will more likely present anterior rotation on this side. Such findings regarding the rotation and/or inclination of the iliac bones are consistent with Beaudoin et al. 1999 [26] who, after studying the acute effects of inducing an LLD by applying a 15mm heel lift alternatively under the right or left foot, reported that a right elevation induced a retroversion of the right iliac bone and an anteversion of the left iliac bone; as opposed to a left elevation which induced a retroversion of the left iliac bone and an anteversion of the right iliac bone. Our results confirm that this phenomenon also occurs for long term natural LLD connected with loading pattern as described above. Our research also found a statistically significant increase in lumbar lordosis for males as a result of wedge correction, while for females there was no change. This indicates that LLD equalisation exerts a greater effect towards change in the lumbo-pelvic girdle for males as compared to females.

One further consideration relates to the comparison (t-Test α = 5%) between the results of the present research and the preliminary results of an earlier research project [47] which however was limited to a subgroup of the postural parameters under consideration here (specifically TKA, LLA, ASO and CA1). The prior research considered a sample of 60 subjects (30 Males and 30 Females) in the same age range and BMI as the present sample; and we have found no statistically significant differences in any of the parameters under consideration, as between the previous and the present research outcomes.

This confirms the validity of the approach and the results presented in this paper and in particular of the signal-processing algorithms for the extraction of clinical parameters. Moreover, it corroborates our proposition that the reported Normative Data represents a stable and reliable set of baseline postural characteristics for any Caucasian population (in our geographic region) with similar physical attributes to our sample [47].

The rapid frame-rate measurement recording system (120 fps in our configuration) seamlessly opens the door to future studies regarding spinal kinematics and segmental coupling during movement such as gait [13,43,48]. Of particular benefit is the fact that in our approach the system can be associated with other measurement systems in a synchronised way, to provide valuable additional data which is not directly clinically available, such as surface electromyography and 3D reaction forces provided by force platforms [10,13,34,35,37,46,48]. Note that the present study used a configuration that included baropodometric measurements for the underfoot loading pattern, without entering into further enquiry about foot/floor interaction and pressure-map characteristics; future studies will involve a deeper and more thorough baropodometric analysis.

### Limitations of the research

As discussed above, the Normative Data presented here describes the full-skeleton upright neutral standing attitude as captured and measured via a 3D opto-electronic stereo-photogrammetric approach; and this is shown to be strongly consistent with the existing data for a healthy young population (with controlled BMI) obtained via other measurement methods, as found in the literature. Nevertheless, there are inherent limitations to the use of cutaneous markers (such as those used here) in different kinds of populations. For example, in cases where there is voluminous subcutaneous tissue the measurements might show a disparity with our results, particularly as regards the distance between the skin-marker and the reference bones on the spine; likewise, the surface profile may diverge from the actual shape of the spine. In addition, for obvious reasons, it is not possible to gather information about vertebra and disc wedging, nor about single-vertebra axial rotations in the case of spinal deformities. Another limitation of this research reflects the present insufficiency of directly-acquired data concerning the upper cervical spine. The data used here has been mathematically reconstructed taking into account the information derived from the position and attitude of the head and from the 3D shape of the thoracic spine, but further studies are necessary to assess its reliability, from a biomechanical-clinical point of view. In our judgement, information derived by placing markers directly on the neck is best avoided if possible, because of the less reliable anatomical relationship between the surface of the back of the neck and the spine. Our view is that such information could lead to excessive (and possibly misleading) approximation in any process of 3D measurement and/or representation.

## Conclusions

This paper presents a stable and reliable set of Normative Postural Data as a reference for evaluating/measuring an upright neutral standing attitude in a young healthy adult population. This data was acquired by means of a novel 3D stereo-photogrammetric approach. It includes a total number of 30 selected 3D quantitative postural, spine morphology and pelvic parameters, which together represent a comprehensive set of standard baseline measurements. These demonstrate a high level of agreement with results obtained via other methods as presented in the existing literature. There is particularly strong consistency with the results, especially in relation to the spine, obtained via X-ray measurements which at this time are the “gold standard”.

The model and the technique described in this study present a number of advantages, specifically their non-invasive aspect, the relatively short time needed for evaluation, and their integration into a complete and coherent postural evaluation. They allow researchers and clinicians to analyse the real, neutral and unconstrained upright standing posture by providing, in a rapid and automatic way, a large number of clinically useful 3D and 2D anatomical/biomechanical/clinical parameters. In addition, all possible postural characteristics are measured simultaneously and at a very fast frame rate while being completely harmless to the subject under examination. These features allow investigators to perform and directly compare multiple different postural tests, obtained within a same-session time frame and in a statistically reliable way.

This paper introduces and describes a first application of the Normative Database, insofar as it has been the basis for comparison between IO and WCO in our sample population. In producing this comparison, the applied methodology demonstrated the ability to accurately capture and represent changes in spinal shape and also to assess the delicate spinal kinematic patterns of individuals in the presence of subtle input, such as the application of an underfoot wedge which was often less than 10mm in size.

Clinical and biomechanical result comparisons, as well as a report output for all 30 quantitative parameters in consideration, are immediately available to both the operator and the subject at the end of acquisition session, the average duration of which is less than 30 minutes. These additional factors further support our approach as being entirely suitable and effective for clinical application. This method was not developed or intended to replace radiographs, from which much more information than spine morphology can be drawn. However, depending on specific clinical purposes, this particular methodology could be used during screening and follow-up to reduce patient irradiation, evaluation time, and cost. This framework is extremely useful as a baseline reference at the diagnostic stage, as well as in designing and developing treatment programs in rehabilitation, in the planning of orthopaedic surgical procedures, and in monitoring the progression of pathology and/or treatment outcomes in any kind of postural disorder and posture-related pathology.

As noted above, to be asymptomatic does not mean to have an optimal posture, and asymmetry was revealed to be a standard for both sexes. Given our results, our system is shown to be a powerful preventative measure. As a rule of thumb, it could be argued that to prevent eventual possible long term negative consequences of asymmetry due to musculoskeletal imbalance on joints and spine, subjects would undergo a fully detailed 3D posture analysis during relevant stages of life, as necessary; and on the basis of this analysis, clinicians could apply a suitable form of LLD equalisation and prescribe regular/periodic performance of focused physical activity, in order to reduce asymmetry in the subject.

## Supporting information

S1 Fig Definition of subject’s anatomical local coordinate system and its relationship to laboratory global coordinate system. (TIF)

S2 Fig Curve angles computation by tangent angles assessment at inflection points. (TIF)

S1 Table Sagittal spine morphology: Agreement between angles computed from profile of spinous processes and angles reported in X-ray based literature. (PDF)

S1 File Raw data source for normative 3D data and statistical analysis. (XLSX)
