# Supplementary figures and images for: Normative 3D opto-electronic stereo-photogrammetric posture and spine morphology data in young healthy adult population
Source: PLoS One. 2017 Jun 22;12(6):e0179619. doi: 10.1371/journal.pone.0179619 (PMC5480974; doi:10.1371/journal.pone.0179619)

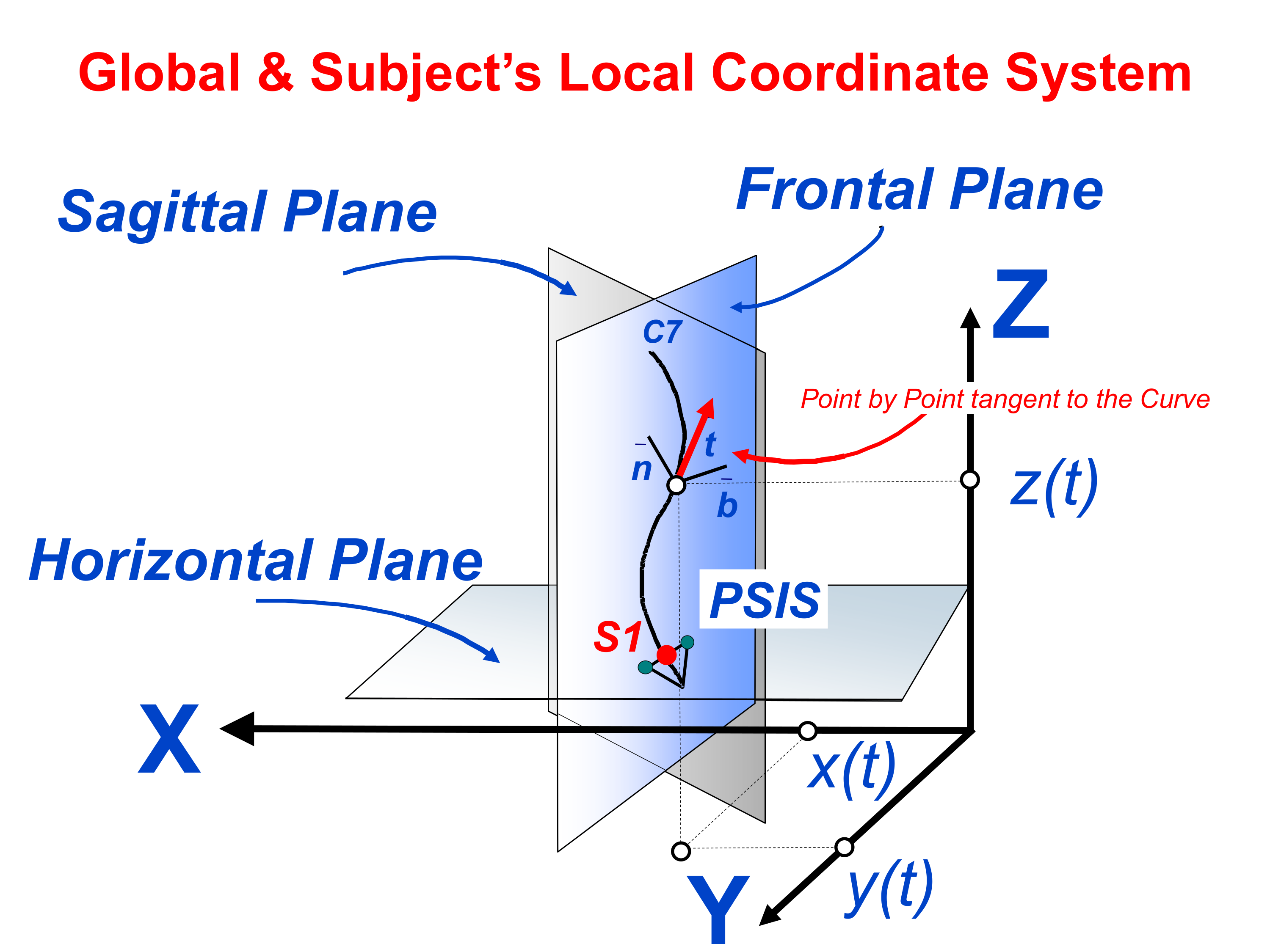

Supplement: S1 Fig — (TIF) [file pone.0179619.s001.tif]

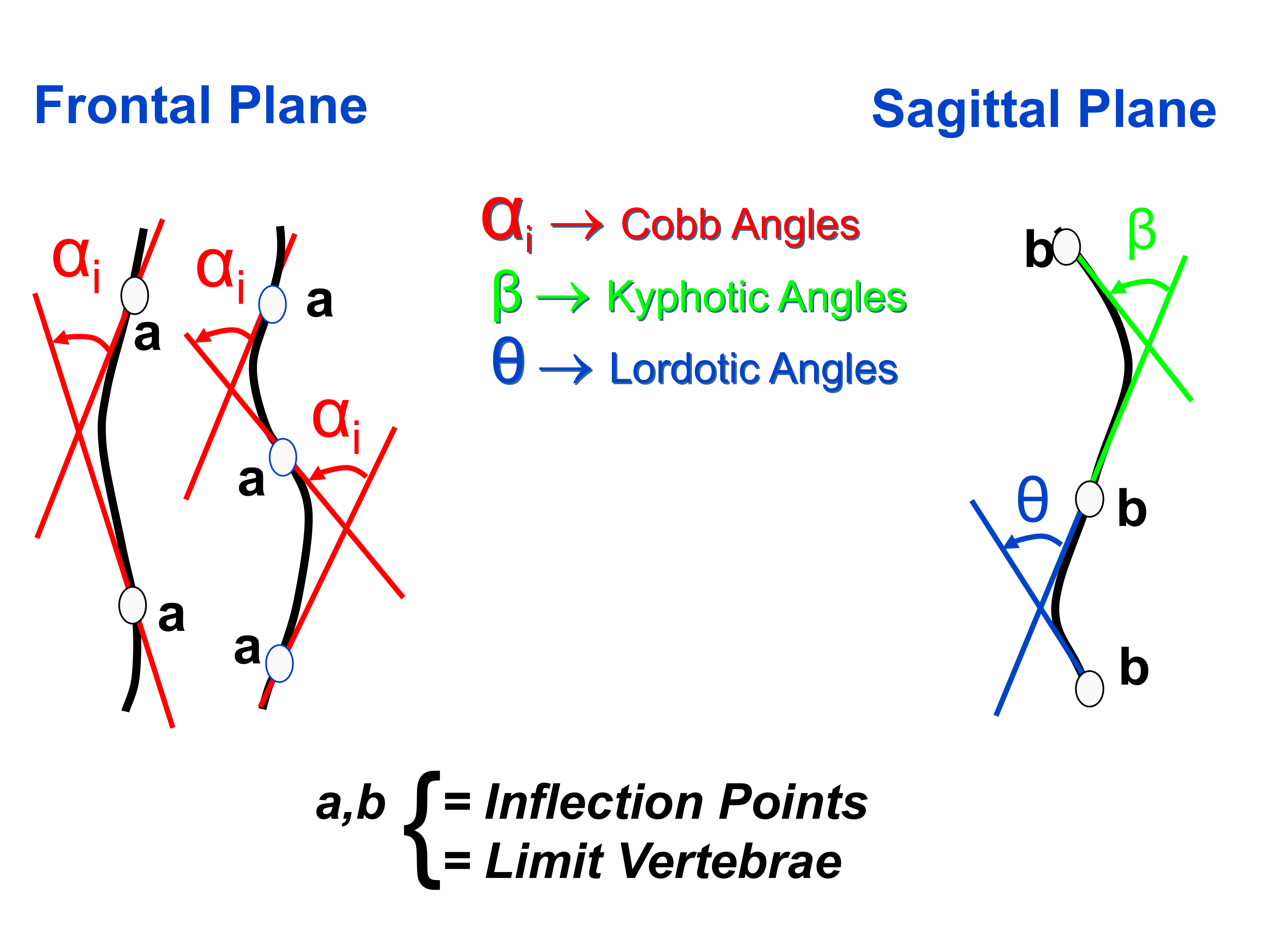

Supplement: S2 Fig — (TIF) [file pone.0179619.s002.tif]
