# Supplementary material for: Normative 3D opto-electronic stereo-photogrammetric posture and spine morphology data in young healthy adult population
Source: PLoS One. 2017 Jun 22;12(6):e0179619. doi: 10.1371/journal.pone.0179619 (PMC5480974; doi:10.1371/journal.pone.0179619)
Supplement: S1 Table — (PDF) [file pone.0179619.s003.pdf]

S1 Table. Sagittal Spine Morphology: Agreement between Angles Computed from Profile of Spinous Processes and Angles Reported in X-ray Based Literature.

**KYPHOSIS**	**Arm position**	**Position along the Spine and n. of included Vertebrae**	**Average ± SD**	**Range Min-Max**
D'Amico et al. (current study) (n = 124)	Free neutral along the side of trunk	TKA	45.98°±8.75°	25.2° – 76.3°
		T1-T12	42.56±8.89	15.0° – 64.8°
		T4-T12	31.9±7.65	14.2° – 51.6°
		KCL (n. of Vertebrae)	10.25±1.11	7 – 14
Amabile et al. 2016 [74] (n = 69) T1-T12	Hands resting on the mandibles	T1-T12	49°±13°	N.A.
Amabile et al. 2016 [74] (n = 69) T4-T12		T4-T12	35.1°±11.5°	N.A.
Roussouly et al. 2005 [94] (n=160)	Hands are placed on rests humerus about 45° with respect to trunk	T1 - Lower Inflection Point	46.4°	22.5° - 70.3°
Vialle et al. 2005 [71] (n = 300)	Arms flexed forward to 45° and resting on support	T4 - T12	40.6°±10°	N.A.
		T4 - Lower inflection point	41.2±10°	N.A.
Fon et al. 1980 [73] (n =37)	Arms above the shoulder in the lateral view	T1 - T12	26.27° ± 8.12°	13° - 48°
**LORDOSIS**	**Arm position**	**Position along the Spine and n. of included Vertebrae**	**Average ± SD**	**Range Min-Max**
D'Amico et al. (current study) (n = 124)	Free neutral along the side of trunk	LLA Males	32.8±8.09	12.5° – 54.8°
		LLA Females	44.20±9.66	24.3° – 68.3°
		L1-L5 Males	25.65°±9.38°	4.0° – 53.0°
		L1-L5 Females	39.45±8.97	16.9° – 60.0°
		LCL Males (n. of Vertebrae)	7.9±1.34	5 – 10
		LCL Females (n. of Vertebrae)	6.84±1.36	4 – 10
Vialle et al. 2005 [71] (n = 300)	Arms flexed forward to 45° and resting on support	Upper inflection point - S1 Male	59.2±10.12	N.A.
		Upper inflection point - S1 Female	62±10	N.A.
		L1-L5 Males	41.4±11	N.A.

		L1 - L5 Females	46.2±11	N.A.
Been and Kalichman 2014 [72] (n = 101)	Arms folded on the chest	L1-L5	39.6°	N.A.
		L1- S1	51.3°±10.7°	N.A.
Roussouly 2005 [94] (no gender separation) (n = 160)	Hands are placed on rests humerus about 45° with respect to trunk	Upper inflection point - S1	61.43°±9.72°	N.A.
		LCL (n. of Vertebrae)	4.5 ± 0.9	1.5 - 7.5
Korovesiss et al. 1998 [91] (n = 99)	N.A.	L1-L5	45.7°	N.A.
Cheng et al. 1998 [96] (n = 387)	N.A.	L1-L5	41.95°	N.A.
Tüzün et al. 1999 [101] (n = 150)	N.A.	L1-L5	45.85°	N.A.
Guigui et al. 2003 [102] (n = 250)	N.A.	L1-L5	43°	13.6° - 69°
		L1- S1	59°	N.A.
Damasceno et al. 2006 [89] (n = 350 no gender separation)	Arms laid on a support in front of the body	L1-L5	45.1°	15° - 78°
		L1-S1	60.9°	33° - 89°
Amabile et al. 2016 [74] (no gender separation) (n = 69)	Hands resting on the mandibles	L1 - L5	46.4°±11.9°	N.A.
		L1- S1	57.6°±12.8°	N.A.
Jackson and McManus, 1994 [87] (n = 100)	N.A.	L1-S1	60.9°	31° - 88°
Jackson et al. 1998 [100] (n = 50)	N.A.	L1- S1	62.1°	41° - 86°
Tsuji et al. 2001 [103] (n = 489)	N.A.	L1-S1	54.2°	N.A.

N.A. = Not Available
